# Management of a giant lymphocele following varicose vein surgery: a case report

**DOI:** 10.1186/s13256-023-04016-7

**Published:** 2023-06-27

**Authors:** Noshi Bibi, Ehtesham-Ul-Haq, Arsh Zahoor, Haroon-Ur-Rashid, Farhan Eitezaaz, Ali Azeem

**Affiliations:** 1 Bahria International Hospital, Bahria Town Phase 8, Rawalpindi, Pakistan; 2 Islamabad, Pakistan; 3 Plastic Surgery Department, Bahria International Hospital, Bahria Town Phase 8, Rawalpindi, Pakistan; 4 Plastic Surgery Department, Birmingham Children’s Hospital, Birmingham, UK

**Keywords:** Lymphocele, Varicose vein, Surgical flaps, Gracilis muscle, Sclerotherapy

## Abstract

**Background:**

A lymphocele or lymphocyst is formed when lymphatic fluid accumulates in a space, following disruption of lymphatic channels. Here, we report a case of a giant lymphocele in a middle-aged female, who underwent Trendelenburg operation (saphenofemoral junction ligation) for varicose veins of her right lower limb.

**Case presentation:**

A 48-year-old Pakistani Punjabi female presented to the plastic surgery outpatient department with a history of painful, progressive swelling of the right groin and medial aspect of the right thigh for 4 months. After investigation, it was diagnosed as a giant lymphocele. A pedicled gracilis muscle flap was used to reconstruct and obliterate the cavity. There was no recurrence of the swelling.

**Conclusion:**

Lymphocele is a common complication after extensive vascular surgeries. In the unfortunate case of its development, prompt intervention must be done to prevent its growth and ensuing complications.

## Background

A lymphocele or lymphocyst [1] is formed when lymphatic fluid accumulates in a space following disruption of lymphatic channels. It occurs commonly in the pelvic, inguinal, and axillary region due to dissection of lymph nodes, renal transplantation, and extensive vascular surgery [1, 2]. Small lymphoceles are mostly asymptomatic and undergo spontaneous resolution. Large lymphoceles, however, begin to produce symptoms due to compression of the surrounding structures. Compression of blood vessels is especially dangerous as it leads to venous stasis and increases the risk of thrombosis. Secondary infections may also occur, leading to an increase in pain severity [3]. Lymphoceles may develop after surgical procedures performed for the treatment of varicose veins or revascularization of the lower limbs [4]. Here, we report a case of a giant lymphocele in the groin region of a middle-aged female after undergoing Trendelenburg’s operation with stripping and multiple stab avulsions for varicose veins.

## Case presentation

A 48-year-old Pakistani Punjabi female presented to the plastic surgery outpatient department (OPD) in March 2020, with a history of painful, progressive swelling of her right groin and medial aspect of the right thigh for 4 months. After detailed history, she had a positive history for deep venous thrombosis (DVT), for which she was on oral anticoagulant therapy. She had undergone Trendelenburg operation (saphenofemoral junction ligation) with stripping and multiple stab avulsions for varicose veins of the right lower limb a year ago. She developed slow growing swelling of her right groin; furthermore, she underwent multiple sessions of aspiration along with compression bandaging. Sclerotherapy was also attempted by her primary operating surgeon, three sessions of 2G tetracycline and one session of 60 mg of bleomycin. These treatment modalities temporarily alleviated the condition but ultimately failed. She is a known patient with diabetes and hypertension, controlled with the oral medication.

**Fig. 1:**
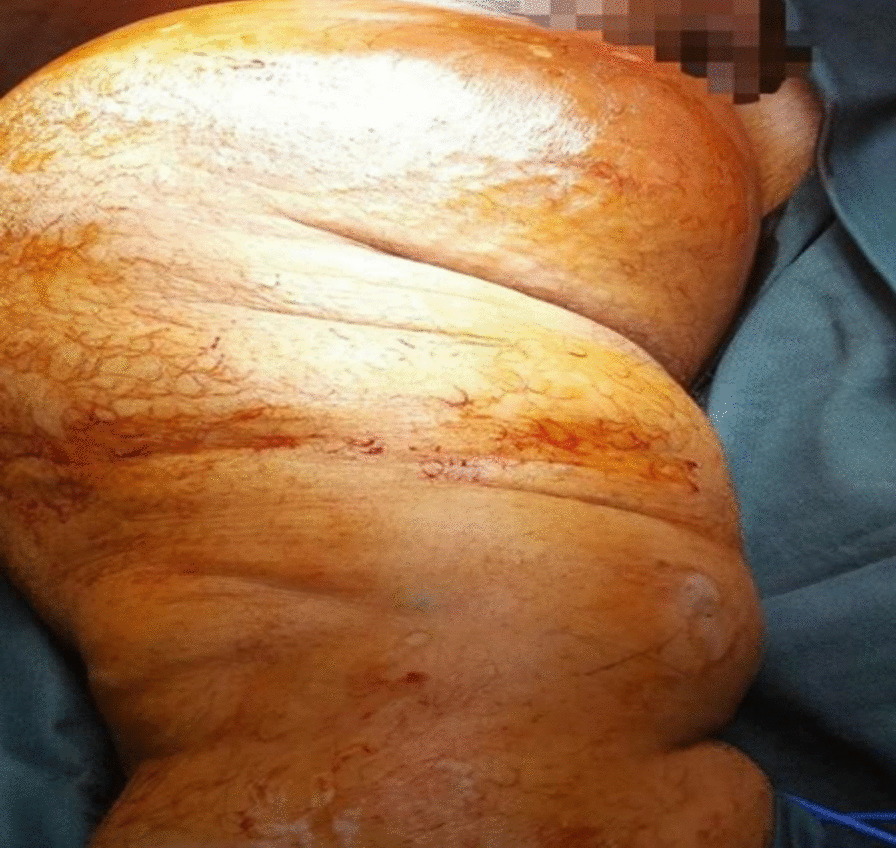
Preoperative image showing the giant lymphocele

On examination, there was a large swelling in the right groin and medial aspect of the right thigh, approximately 65 cm × 25 cm. It was tense, cystic, and slightly tender on palpation. The overlying skin was warm and appeared to be red and shiny. A long transverse scar was also seen in the groin, along with multiple smaller scars over the whole lower limb, which was a sign of her previous surgery for varicose veins. The girth of the right thigh was 96 cm, measured at the time of initial presentation, and currently, in her last follow-up, it was 73 cm compared with the 65 cm girth of her other thigh. Her rest of the systemic examination was unremarkable (Fig. 1).

Routine investigations were carried out. Her complete blood count (CBC) showed a raised white blood cell count of 22,000 with neutrophils dominating (82%). The rest of the parameters were within normal limits. Her glycosylated hemoglobin (HbA1c) levels were raised at 8.2%, signifying poor control of glucose levels. Her liver function test was normal and hepatitis profile was negative. Needle aspiration was also carried out. The fluid was found to be straw colored and was sent for routine analysis and cytology.

Magnetic resonance imaging (MRI) was the mainstay for diagnosing her condition, which showed a large thin-walled hypoechoic lesion. The swelling was therefore diagnosed as a case of a refractory giant lymphocele and the patient was admitted and optimized for surgery. Under general anesthesia, the patient was placed in a supine frog leg position for ease of surgical site access. Subdermal methylene blue dye injections were given at four points around the swelling in the region of the thigh and anterior abdominal wall. This procedure was performed to observe the absorption of the dye, which would help locate any lymphatic vessels draining into the cystic cavity.

**Fig. 2:**
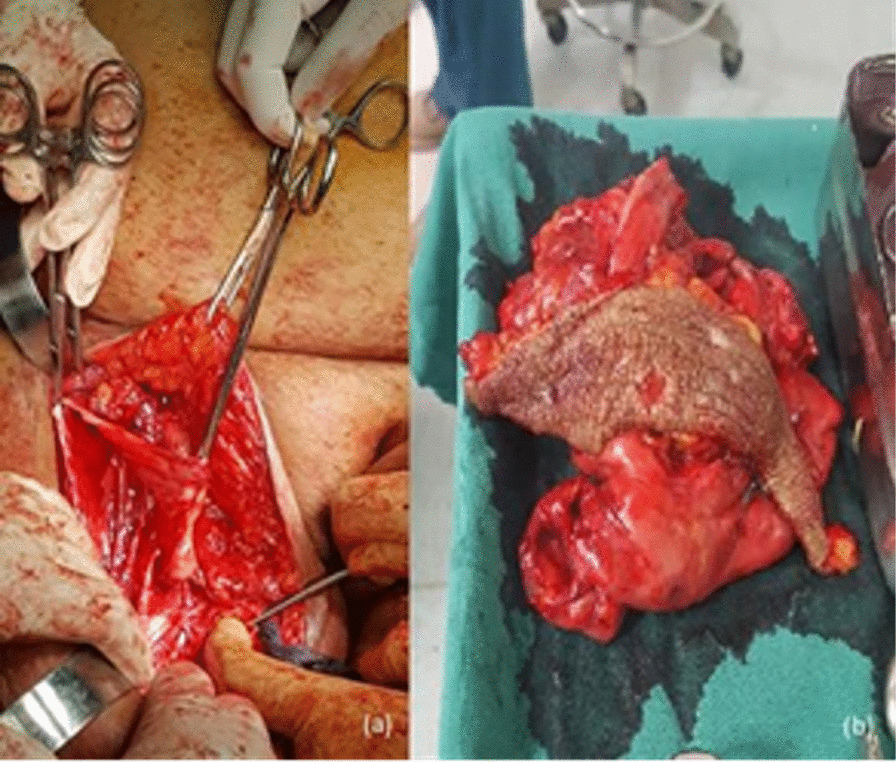
**a** Intraoperative image showing the lymphocele cavity. **b** Excised transverse groin scar and pseudo bursa

**Fig. 3:**
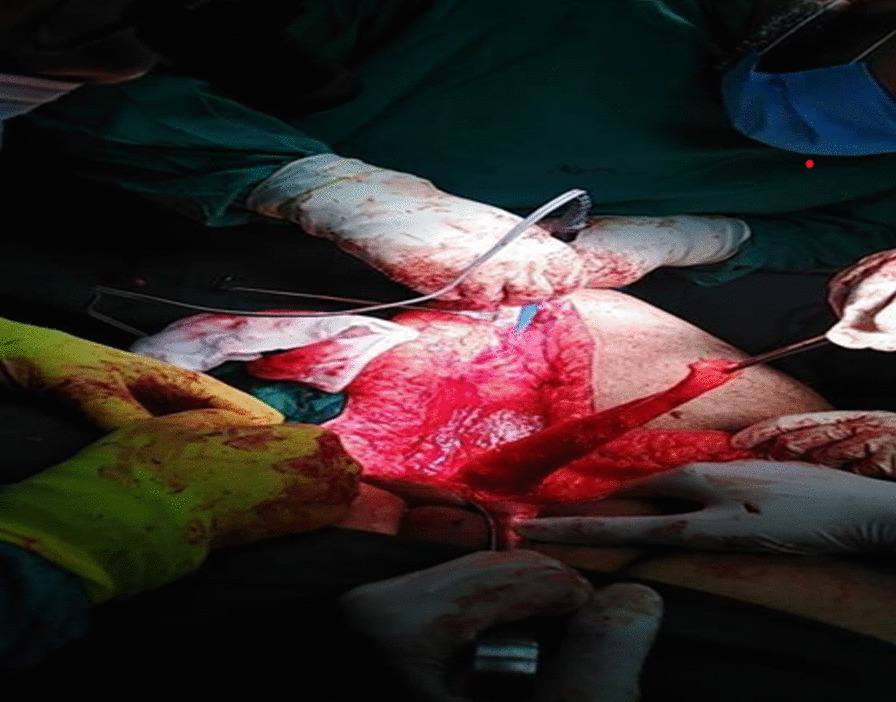
Intraoperative image showing the harvested pedicle gracilis muscle flap

**Fig. 4:**
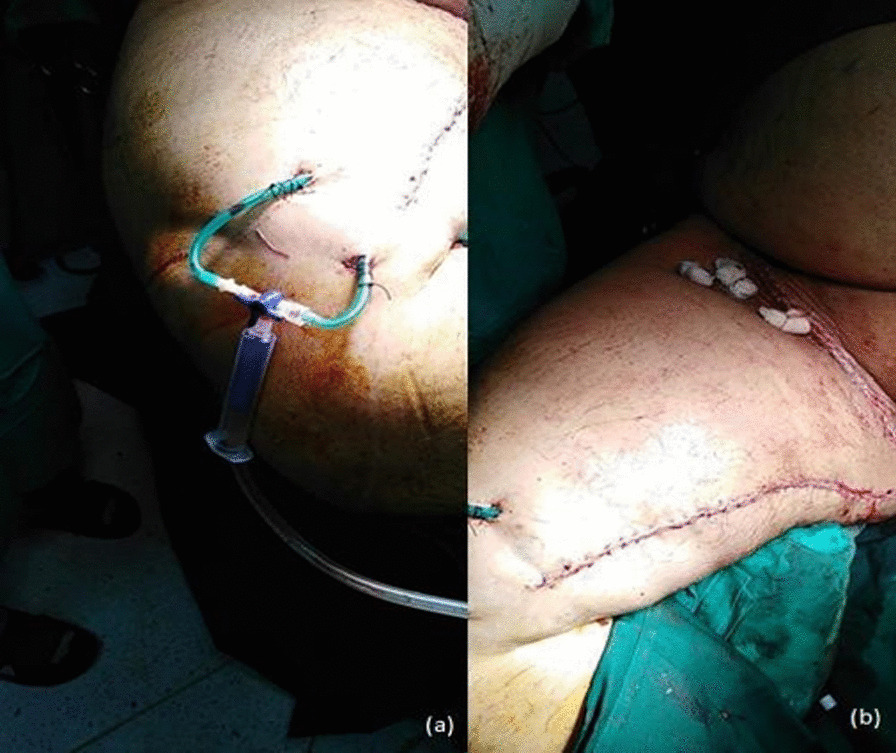
Image showing the placement of surgical drain (**a**) and bolster (**b**)

A stab incision was made at the convexity of the swelling and 6 L of yellow straw-colored fluid was drained from it. A transverse incision was made over the swelling, utilizing the groin scar already present. The scar was excised and the cavity was explored. The methylene blue dye was not encountered in the cyst cavity during exploration. Excision of the pseudo bursa was performed (Fig. 2). However, a small portion of the bursa wall overlying the femoral triangle was left *in situ* as there was a fear of damaging the femoral vessels due to the presence of dense adhesions. It was gently curetted to make it rough and for it to bleed to promote the formation of adhesions. A pedicled Gracilis muscle flap was raised and rotated and inserted transversely to obliterate the resultant cavity (defect), with the expectation of promoting lymphatic drainage (Fig. 3). It was secured using Vicryl 2/0 sutures. Hemostasis was secured. The dead space was then further eliminated with the help of quilting sutures. Two drains were placed, and the overlying skin was stabilized with bolster sutures to prevent its separation due to lymphatic fluid collection (Fig. 4). The drainage tubes were connected to a low vacuum suction, which was turned on for 20 minutes and then off for an equal amount of time. The cycle was repeated throughout the postoperative hospitalization period of the patient.

**Fig. 5:**
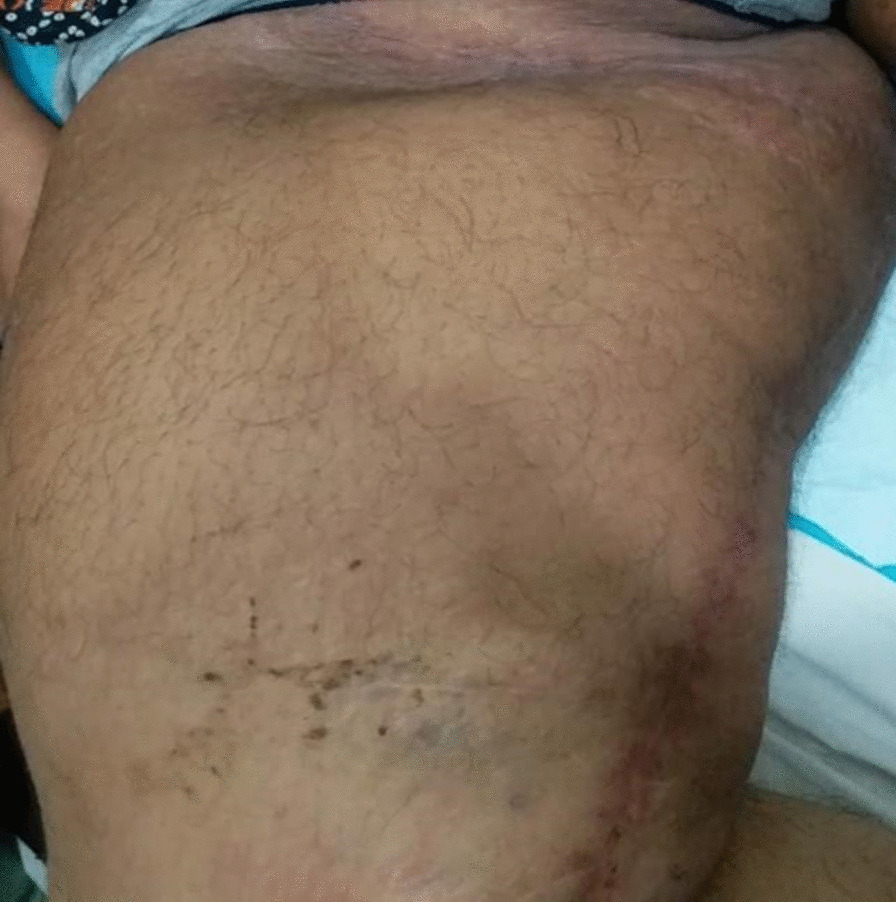
Follow-up image after 1 year with complete resolution of lymphocele

**Fig. 6:**
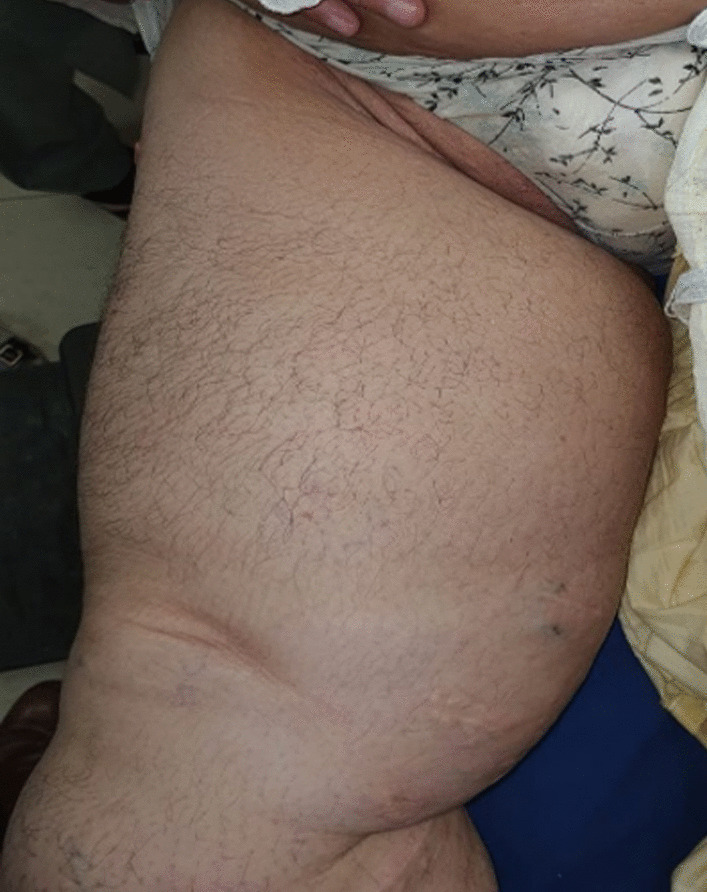
Follow-up after 2 year showing no recurrence of lymphocele

Drains were removed at 2 weeks and the patient was discharged on oral prophylactic antibiotics and analgesics. Skin holding bolsters were not removed for another 4 weeks. The patient returned for follow-up after 6 weeks and a small collection was noted, measuring 7 cm × 5 cm × 3 cm on the medial side of the thigh. It was aspirated and a pressure dressing was applied, which led to its resolution. On each of the follow-up visits that were scheduled every 3 months over a year, bilateral lower limb girth was measured. The measurement of the right thigh was almost the same as for the left thigh. Fortunately, the lymphocele had completely resolved and no further recurrence of the swelling was detected, and her follow-up is done up to 2-years postoperative. (Figs. 5, 6).

## Discussion

Iatrogenic lymphatic leakage is a common complication of extensive surgery, with the development of a lymphocele occurring within 3–8 weeks or 1 year mostly due to lymph node dissection, pelvic surgery and renal transplantation. Groin lymphoceles are also found to occur after reconstructive surgery involving the femoral artery in around 1.5%–8% patients [5]. Other risk factors include neoadjuvant radiotherapy, heparin prophylaxis, and involvement of the lymph nodes by tumor cells [6]. The aim of this case report in particular was to describe lymphocele formation with intact lymph nodes and treatment with pedicle gracilis flap, functioning as a drainage channel that led to outflow of lymphatic fluid, which ultimately lead to the resolution of symptoms with no recurrence and has shown great results long term.

Obesity is a known risk factor that leads to formation of primary lymphedema. In primary lymphedema, either the channels are obliterated or become narrow because of the adipose deposition and chronic inflammation around the lymphatic channels. Diabetes and obesity lead to gradual formation of lymphedema and can be treated early with weight loss intervention, good glycemic control before the onset of irreversible lymphatic dysfunction. Considering that substantial weight loss is a difficult and time consuming task for patients, lymphaticovenous anastomosis (LVA), in combination with non-surgical intervention, is a good option for these patients [7]. Such treatment is beneficial in patients with no history of heart failure, renal failure, deep vein thrombosis, and chronic venous obstruction or insufficiency. In our patient, she was suffering from varicose veins due to chronic insufficiency and had superficial stripping of the dilated veins and ligation at main branches of groin by the primary surgeon. Due to extensive dissection at saphenofemoral junction, it disrupted the lymphatic channels and was the cause of lymphocele formation. She was not a candidate for lymphovenous bypass because of the stripping of superficial veins along the entire affected lower limb.

Intraoperative measures are taken to reduce the occurrence of a lymphocele. These include meticulous control of the divided lymphatic channels, with the help of electrocautery, or by ligation with sutures or clips. Peritonization is also a technique performed, in which the retroperitoneum is left open after surgery to allow drainage of lymphatic fluid and prevent its accumulation. This is considered the gold standard technique of prevention [8]. Placement of a surgical drain has also shown to decrease the chances of lymphocele formation [9].

Majority of the lymphoceles are small in volume and therefore produce none or few signs and symptoms and resolve spontaneously. Larger lymphoceles, however, produce various symptoms, depending upon its site and by the compression of surrounding structures.

Management of such lymphoceles is done by both conservative and surgical techniques. Conservative management includes compression bandaging and limb elevation to improve lymph flow and drainage, prophylactic antibiotics to prevent lymphocele infection, and closed drainage of the lymphocele via needle aspiration or by catheter insertion and drainage [6].

Sclerotherapy is a commonly practiced procedure, which includes the instillation of a sclerosing agent, such as ethanol and povidone iodine, into a cavity to cause fibrosis and tissue contraction, which therefore causes the cavity to collapse. It has high affectivity, especially when performed along with aspiration or drainage, and has a recurrence rate of around 20%–40%, decreasing according to the number of sessions. However, in cases of large refractory lymphoceles in which the fluid volume exceeds 500 mL, such as in our case, sclerotherapy has not shown to be effective [10]. The primary surgeon tried multiple sessions of aspiration and sclerotherapy; however, there was recurrence of the lymphocele and the patient was thus referred to us for the definitive management. Sclerotherapy in such a large lymphocele was not effective, rather it aggravated the symptoms.

Failure of resolution with conservative management, or large lymphoceles that are not expected to resolve only with conservative methods, as in the case we presented, calls for surgical intervention. Surgical procedures include lymphocelectomy and fenestration and marsupialization, either open or laparoscopic, lymphovenous bypass, or combination of lymphovenous pass and sclerotherapy for large lymphoceles. In combined therapy, the main function of sclerotherapy is to close the residual dead space and prevent formation of an empty space in which fluid can again easily accumulate, furthermore the lymphovenous bypass is done to divert lymph flow and prevent the recurrence of the disease. The success rate of sclerotherapy was directly related to the size of lymphocele cavity, with larger cavities being related to a lower probability of success and warranting an additional surgery to establish the lymphatic flow [11]

Lymphovenous bypass is particularly indicated for mild-to-moderate conditions, where the swelling is not too severe. When the amount of accumulated fluid is limited, it is sufficient to perform one or more LVA, but when the volume becomes bigger, this is no longer sufficient as a large dead space inside the capsule remains and spontaneously tends to be filled with interstitial fluid. In our case, due to stripping of the veins, this combined therapy was not advisable as there would not be any superficial vein for anastomosis. For this reason, we integrated the lymphatic treatment with a pedicled gracilis muscle flap. In this way we were able to properly fill the dead space generated by the lymphocele. This is a very important point since a good restoration of the volumes prevent the formation of empty spots that might facilitate recurrences. To conclude the main function of the muscle flap was just to fill the dead space and reconstruct the missing volume, preventing the formation of an empty space where fluid could easily accumulate [12]. A newer technique known as “packing of the groin” was also found to be beneficial in cases of groin lymphocele. This technique involves suturing plastic pellets around the lymph nodes, leading to their compression by the soft tissues [13]. In our case, lymphocelectomy was done with excision of the pseudo bursa, followed by a pedicle garcilis muscle flap to obliterate the cavity and resolve the lymphocele. Two years after her surgery there is no recurrence and she is carrying out her daily activities without any discomfort.

## Conclusion

Groin lymphoceles are a common postoperative complication of extensive groin surgery, with an incidence of 1%–87% [14]. Appropriate measures should be taken intraoperatively, as well as postoperatively, to reduce the risk of its formation. In the unfortunate case of its development, prompt intervention must be done to prevent its growth and ensuing complications. Excision of the pseudo bursa plays an important role in the surgical management of lymphoceles. If not performed, the chances of recurrence increase. Obliteration of dead space with the help of a muscle flap, especially of the gracilis muscle, has been proven to be a definitive treatment modality in the groin region. It is also found to improve lymphatic drainage by the formation of new lymphatic channels^.^

## Data Availability

Data cannot be shared because of institution does not allow sharing of data and materials.
